# IL-37b alleviates endothelial cell apoptosis and inflammation in Kawasaki disease through IL-1R8 pathway

**DOI:** 10.1038/s41419-021-03852-z

**Published:** 2021-06-03

**Authors:** Chang Jia, Yingzhi Zhuge, Shuchi Zhang, Chao Ni, Linlin Wang, Rongzhou Wu, Chao Niu, Zhengwang Wen, Xing Rong, Huixian Qiu, Maoping Chu

**Affiliations:** 1 Pediatric Research Institute, The Second Affiliated Hospital and Yuying Children’s Hospital of Wenzhou Medical University, 325027 Wenzhou, China; 2 Children’s Heart Center, Institute of Cardiovascular Development and Translational Medicine, The Second Affiliated Hospital and Yuying Children’s Hospital of Wenzhou Medical University, 325027 Wenzhou, China

**Keywords:** Vasculitis, Drug development

## Abstract

Kawasaki disease (KD) is an acute vasculitis of pediatric populations that may develop coronary artery aneurysms if untreated. It has been regarded as the principal cause of acquired heart disease in children of the developed countries. Interleukin (IL)-37, as one of the IL-1 family members, is a natural suppressor of inflammation that is caused by activation of innate and adaptive immunity. However, detailed roles of IL-37 in KD are largely unclear. Sera from patients with KD displayed that IL-37 level was significantly decreased compared with healthy controls (HCs). QRT-PCR and western blot analyses showed that the expression level of IL-37 variant, IL-37b, was remarkably downregulated in human umbilical vein endothelial cells (HUVECs) exposed to KD sera-treated THP1 cells. Therefore, we researched the role of IL-37b in the context of KD and hypothesized that IL-37b may have a powerful protective effect in KD patients. We first observed and substantiated the protective role of IL-37b in a mouse model of KD induced by *Candida albicans* cell wall extracts (CAWS). In vitro experiments demonstrated that IL-37b alleviated endothelial cell apoptosis and inflammation via IL-1R8 receptor by inhibiting ERK and NFκB activation, which were also recapitulated in the KD mouse model. Together, our findings suggest that IL-37b play an effective protective role in coronary endothelial damage in KD, providing new evidence that IL-37b is a potential candidate drug to treat KD.

## Introduction

Kawasaki disease (KD) is an illness that can cause systemic immune vasculitis, which mainly has an effect on young children. The most severe complication of KD is coronary artery injury, including coronary artery dilation, coronary artery aneurysm (CAA) and stenosis. Clinically, the state-of-the-art therapeutic strategy is intravenous immunoglobulin (IVIG) plus aspirin. In the absence of early intervention, about 25% of KD patients would develop CAA. Even with timely treatment, there is still unacceptable high risk of giving rise to CAA. Therefore, it is urgent to explore novel and potential therapeutic strategy to clinically treat KD and its complications.

Interleukin-37 (IL-37) is a newly reported member of the IL-1 family and functions as a novel anti-inflammatory cytokine to suppress inflammatory and immune response. Studies report that IL-37 plays an essential role in mediating inflammatory diseases, including arthritis^1^, liver inflammation^2^, and cardiovascular disease inflammation^3^. In addition, increasing evidence demonstrate that exogenous IL-37 can ameliorate cardiovascular injury, including vascular calcification, atherosclerosis^4^, aortic valve lesion^5^, myocardial infraction^6^, and myocardial ischemia/reperfusion injury^7^. Currently, five splice variants of human IL-37 (IL-37a–e) have been identified, with IL-37b being the largest and most investigated. Extracellular IL-37 and its splice variants IL-37b and IL-37d are reported to mitigate inflammation via binding to IL-1R8^3,8–12^. IL-1R8, also termed as single immunoglobulin G (IgG) IL-1-related receptor, has merely a single Ig domain and is able to provide a brake on inflammation^13^. Presently, whether KD vasculitis can be ameliorated by IL-37 and its subtypes is still unidentified.

As is known, endothelial cell (EC) injury and inflammation are the key pathological mechanisms for KD, and alleviating endothelial injury or inflammation can be an effective mean to treat KD^14–16^. IL-37 exerts anti-inflammatory response in many types of cells, including ECs^17^. Recent studies revealed that IL-37 inhibit inflammatory response in human coronary artery ECs via intracellularly suppressing the Toll-like receptor 2-nuclear factor κB (NFκB)-intercellular adhesion molecule 1 (ICAM-1) pathway^17^. In addition to anti-inflammatory activity, IL-37 is also related to apoptosis. For instance, Li et al. reported that IL-37 attenuates lipopolysaccharide (LPS)-induced neonatal acute respiratory distress syndrome in young mice via inhibiting inflammation and cell apoptosis^18^. Wu et al. demonstrated that mice treated with IL-37 show an obvious amelioration of the ischemia/reperfusion injury, as demonstrated by reduced infarct size, decreased cardiac troponin T level, and improved cardiac function. This protective effect was associated with the ability of IL-37 to suppress production of pro-inflammatory cytokines, chemokines, and neutrophil infiltration, which together contributed to a decrease in cardiomyocyte apoptosis and reactive oxygen species generation^7^. Until now whether IL-37 can inhibit EC apoptosis, especially in KD, remains still unknown.

To investigate the effect of IL-37 on KD, we first examined the level of IL-37 in the serum from KD patients as compared with healthy controls (HCs) and affirmed that its splice variant IL-37b might function in KD. Then we investigated the therapeutic effect of IL-37b on KD through in vivo and in vitro experiments. Ultimately, we substantiated that IL-37b alleviated KD through IL-1R8 pathway by inhibiting the activation of extracellular signal–regulated kinase (ERK) and NFκB. This study may provide a potentially therapeutic target for KD coronary endothelial injury.

## Results

### IL-37b expression level was significantly decreased in KD serum-treated human umbilical vein endothelial cells (HUVECs)

**Fig. 1:**
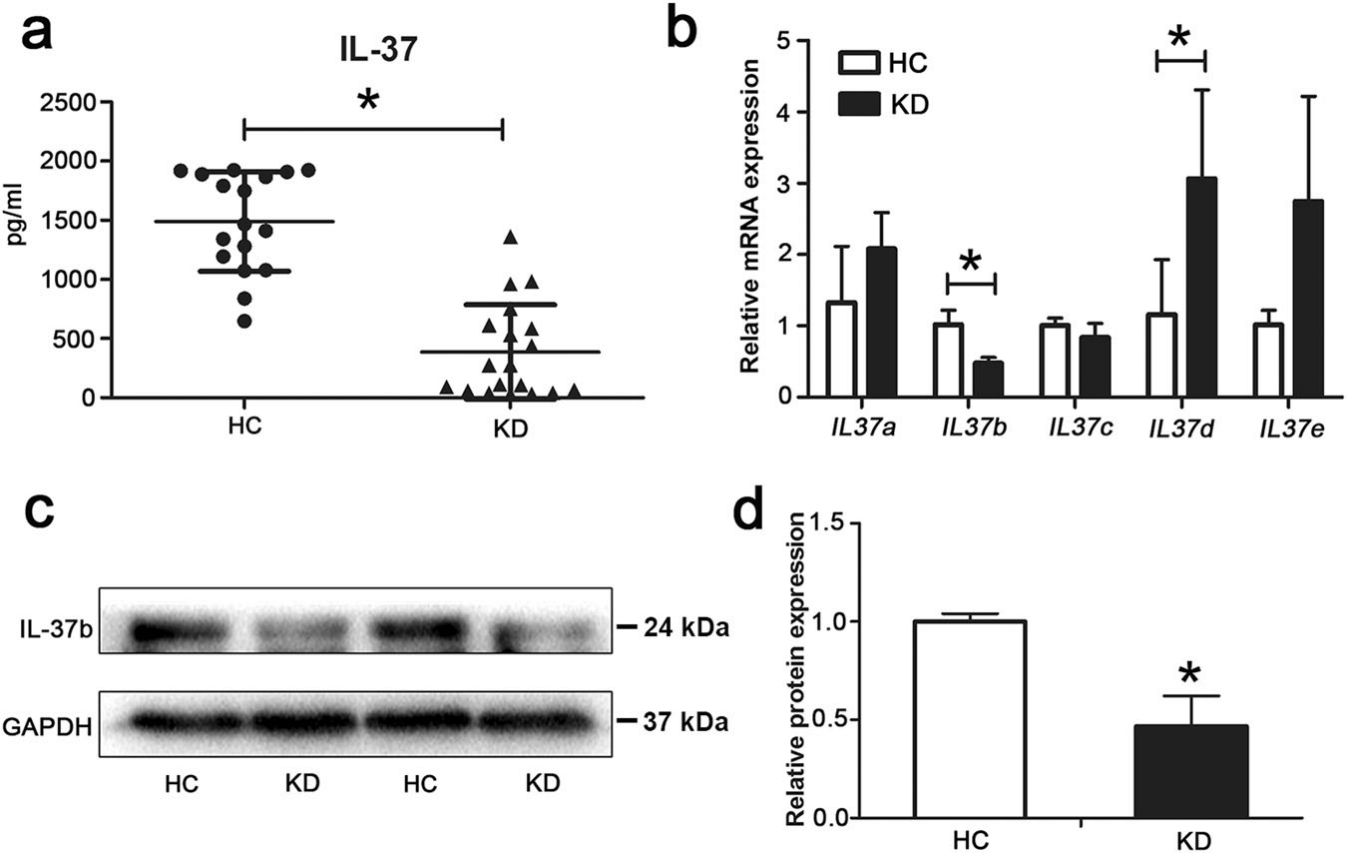
The expression level of IL-37b was decreased after KD sera treatment. **a** The levels of IL-37 in sera from health controls (HCs, *n* = 17) and KD patients (*n* = 18) were evaluated by enzyme-linked immunosorbent assay (ELISA). Significance: *P < 0.05. **b** The mRNA expression levels of five IL-37 splice variants (IL-37a–e) were evaluated in KD-treated ECs by qRT-PCR analysis. Significance: *P < 0.05. **c**, **d** Protein expression of IL-37b was assessed in KD-treated ECs by western blot analysis. GAPDH was used as an internal control. Quantitative analysis of IL-37b expression was performed (**d**). All these experiments were repeated at least three times. Data are shown as mean ± SD (*n* = 3). Significance: *P < 0.05.

To preliminarily confirm that IL-37 might be involved in the pathological process of KD, we first examined the level of IL-37 in the serum from KD patients compared with the HCs. Enzyme-linked immunosorbent assay (ELISA) showed that the serum level of IL-37 was much lower in KD patients (Fig. 1a). Next, the mRNA expression levels of five splice variants of *IL37* gene, *IL37a–e*, were determined in KD serum-treated ECs using quantitative real-time polymerase chain reaction (qRT-PCR) analysis. Results showed that only *IL37b* was significantly downregulated in KD serum-treated ECs compared with HC serum-treated ECs (Fig. 1b). Western blot analysis further substantiated that the protein expression level of IL-37b was decreased after KD serum treatment (Fig. 1d). Taken together, the expression of IL-37b was downregulated in ECs after KD serum treatment.

### Recombinant human IL-37b alleviates coronary artery injury in a KD mouse model

**Fig. 2:**
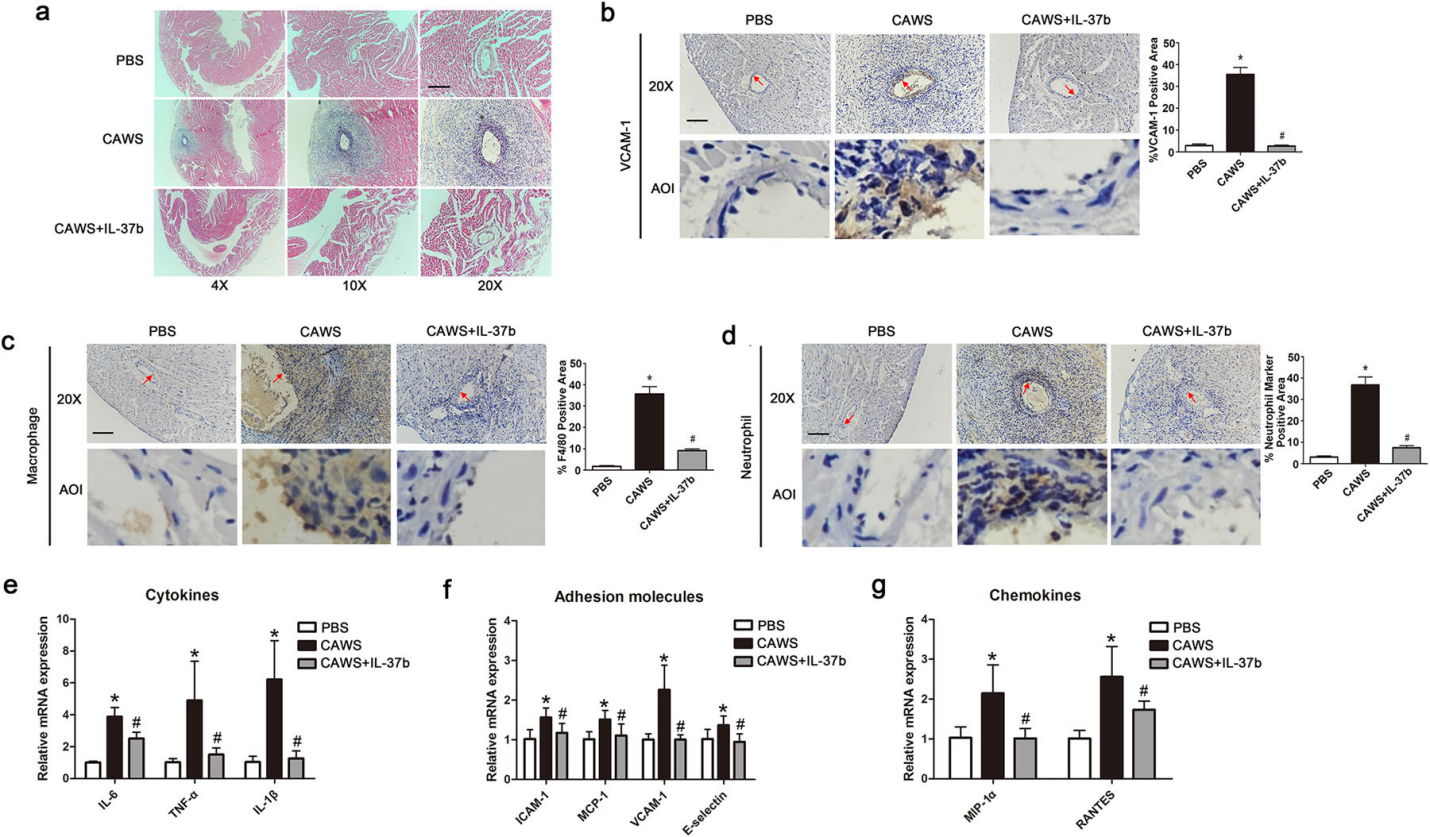
The coronary arteritis was mitigated after Il-37b injection in a KD mouse model. **a** Representative images from H&E staining at 28-day post-CAWS injection. Magnification: ×200. Scale bar = 100 μm. **b**–**d** The expression levels of vascular cell adhesion molecule 1 (VCAM-1), macrophage marker F4/80, and neutrophil marker were, respectively, determined using IHC staining in the endothelium of coronary arteries. Enlarged images of area of interesting (AOI) were indicated with a red arrow. Scale bar = 100 μm. **e**–**g** The expression levels of cytokines, adhesion molecules, and chemokines were detected in the mice heart section. Significance: *P < 0.05 vs. the PBS group, and ^#^*P* < 0.05 vs. the CAWS group. *N* = 6 mice per group, and each experiment was conducted at least three times.

IL-37b has been reported to have potent anti-inflammatory activities in autoimmune diseases^19,20^. However, its function in KD is still unclear. To initially investigate the role of IL-37b, recombinant human IL-37b was intraperitoneally injected into *Candida albicans* cell wall extract (CAWS)-induced KD mouse model. As expected, treatment with IL-37b significantly attenuated coronary artery inflammation (Fig. 2a). As is known, vascular cell adhesion molecule 1 (VCAM-1) is an essential molecule to mediate the adhesion of many leukocytes to the vascular endothelium, the elevated expression of which is regarded as a marker for EC activation during vascular inflammation^21^. As shown in Fig. 2b, immunohistochemistry (IHC) analysis demonstrated treatment of mice with CAWS significantly upregulated the expression of VCAM-1 in the intima of coronary artery, coupled with increased infiltration of inflammatory cells involved in macrophages and neutrophils (Fig. 2c, d). In addition, the expression of inflammation-related factors, including cytokines, adhesion molecules, and chemokines, were also upregulated in the KD mouse model (Fig. 2e–g), consistent with previous studies^22^. However, IL-37b injection remarkably decreased VCAM-1 expression levels, inflammatory cell adhesion, and expression of inflammation-related factors, indicating that IL-37b possesses a good anti-inflammatory effect in KD.

### Recombinant human IL-37b mitigated EC apoptosis and inflammation

**Fig. 3:**
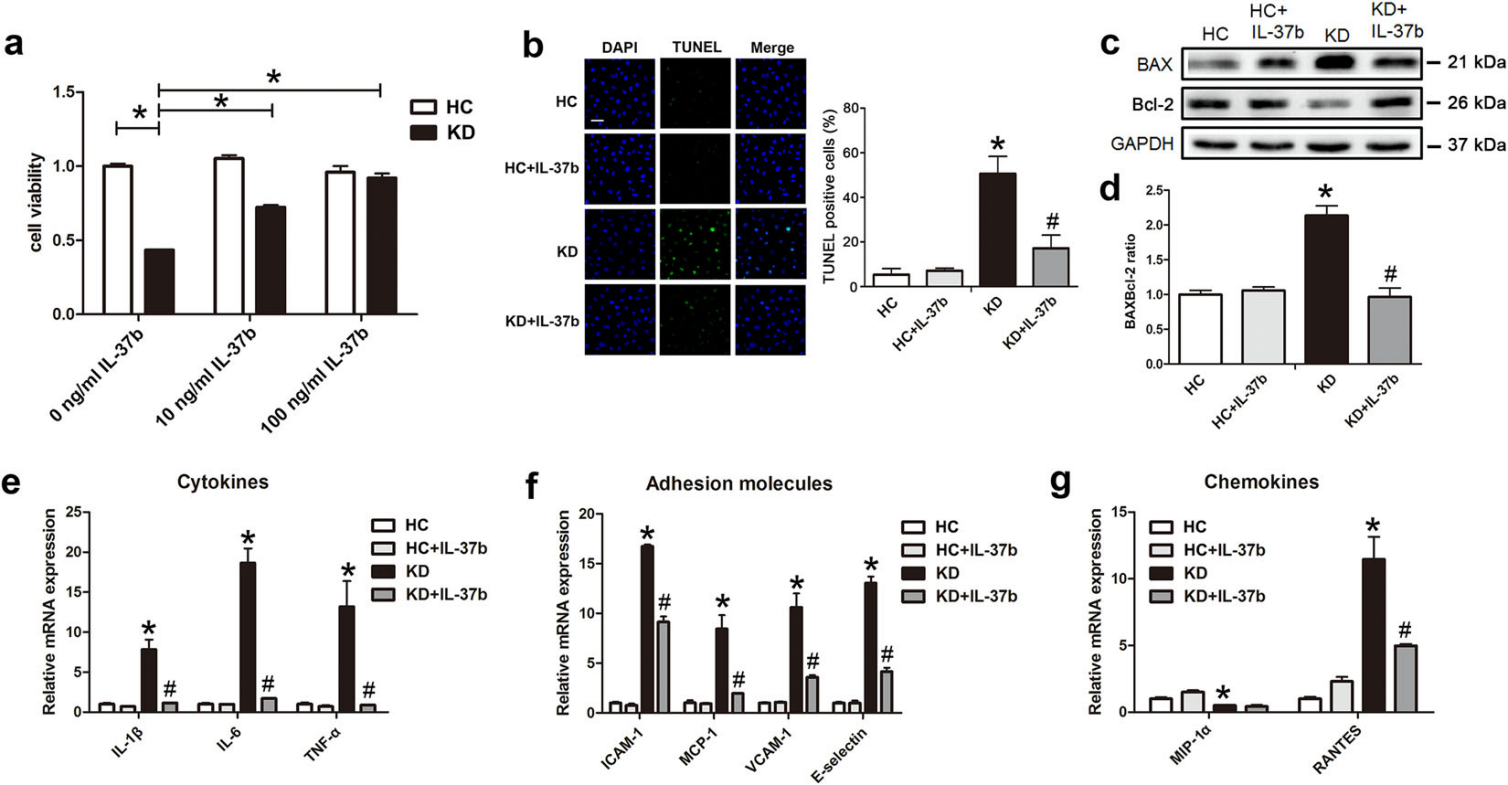
IL-37b alleviated KD-treated endothelial cell apoptosis and inflammation. **a** CCK8 assay was used to evaluate cell viability (*n* = 6). Significance: *P < 0.05. **b** DNA fragmentation was analyzed using TUNEL staining (*n* = 3). **c** Protein expression of BAX and Bcl-2 was determined by western blot analysis. **d** Quantitative analysis of BAX/Bcl-2 ratio was conducted. Data are presented as mean ± SD (*n* = 3). **e**, **f** The expression levels of cytokines, adhesion molecules, and chemokines were examined in KD-treated ECs after IL-37b treatment (*n* = 3). *P < 0.05 vs. the HC group, and ^#^*P* < 0.05 vs. the KD group. At least three experiments were performed for each assay.

Previous studies and the above in vivo results demonstrated that vascular ECs were damaged in KD^21,23^, and IL-37b could alleviate EC injury and the subsequent coronary artery inflammation. Considering ECs are exposed to inflammatory environment in KD, we utilized KD sera-treated THP1 cells and co-cultivated them with HUVECs to create an in vitro coculture experimental system as described by our previous studies^16^. To investigate the protective efficacy of IL-37b, different concentrations of recombinant human IL-37b were added into ECs before co-culturing them with KD sera-treated THP1 cells (referred to as “KD-treated ECs” from now on). Cell Counting Kit-8 (CCK8) analysis exhibited that IL-37b markedly rescued cell viability, and 100 ng/ml IL-37b had the best rescue effect (Fig. 3a). Therefore, 100 ng/ml IL-37b was chosen to treat cells in the follow-up experiments. Studies reported that IL-37 can improve the survival rate of podocytes through suppressing high glucose-induced apoptosis^24^. Therefore, we examined the effect of IL-37b on EC apoptosis. Data displayed that IL-37b significantly inhibited KD sera-induced EC apoptosis, as evidenced by declined percentage of terminal deoxynucleotidyl transferase-mediated dUTP-fluorescein nick end labeling (TUNEL)-positive cells and downregulated BAX/Bcl-2 ratio compared with the KD-treated EC group (Fig. 3b–d). In addition, we found that IL-37b treatment significantly decreased the expression levels of inflammation-related factors in KD-treated ECs, including IL-1β, IL-6, tumor necrosis factor (TNF)-α, ICAM-1, monocyte chemotactic protein-1, VCAM-1, E-selectin, and RANTES (regulated and normal T cell expressed and secreted; Fig. 3e–g). The above data indicate that IL-37b addition can inhibit KD-treated EC apoptosis and inflammation.

### IL-37b-mediated inhibition of EC apoptosis and inflammation was associated with IL-1R8

**Fig. 4:**
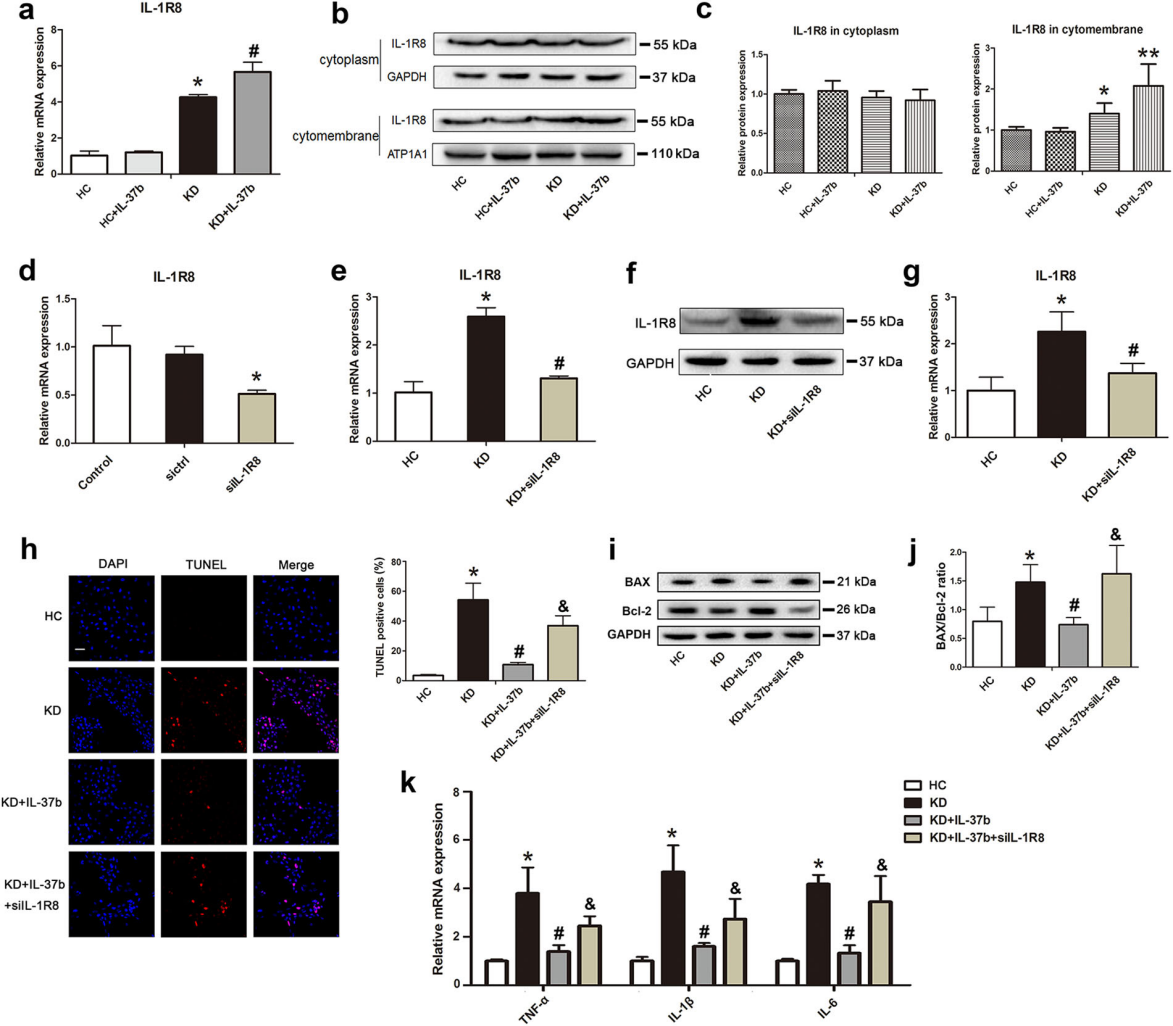
IL-37b alleviated endothelial cell apoptosis and inflammation via IL-1R8 pathway. **a** The mRNA expression of IL-1R8 was examined by qRT-PCR analysis (*n* = 3). Significance: *P < 0.05 vs. the HC group, and ^#^*P* < 0.05 vs. the KD group. **b**, **c** The surface and intracellular expression levels of IL-1R8 were examined by western blot analysis (*n* = 3). GAPDH was used as an internal control of cytoplasmic proteins, and ATP1A1 was utilized as an internal control of cell surface membrane proteins. Significance: *P < 0.05, and **P < 0.01. **d** Endothelial cells were transiently transfected with nontargeting control siRNA (sictrl) or IL-1R8-specific siRNA (siIL-1R8). After that, the expression change of IL-1R8 was examined (*n* = 3). **e**–**g** Endothelial cells that were transiently transfected with siIL-1R8 were treated with KD sera-treated THP1, and then the IL-1R8 expression was determined at the mRNA level (**e**) and protein level (**f**, **g**). *P < 0.05 vs. the HC group, and ^#^*P* < 0.05 vs. the KD group. **h** DNA fragmentation was analyzed using TUNEL staining after IL-1R8 expression was silenced (*n* = 3). Significance: *P < 0.05 vs. the HC group, ^#^*P* < 0.05 vs. the KD group, and ^&^*P* < 0.05 vs. the KD + IL-37b group. Magnification: ×200, Scale bar = 100 μm. **i** Protein expression of BAX and Bcl-2 was determined after IL-1R8 expression was silenced. **j** Quantitative analysis of the ratio of BAX/Bcl-2 performed. Data were expressed as mean ± SD (n = 3). **k** The expression levels of cytokines were assessed after silencing IL-1R8 expression (*n* = 3). Significance: *P < 0.05 vs. the HC group, ^#^*P* < 0.05 vs. the KD group, and ^&^*P* < 0.05 vs. the KD + IL-37b group. All these experiments were done at least three times.

Previous studies reported that extracellular forms of IL-37 function via the IL-1 family decoy receptor IL-1R8^25,26^. To further investigate the mechanism of IL-37b in KD, the mRNA expression level of IL-1R8 was determined. Results showed that treatment with KD serum significantly upregulated IL-1R8 expression, and addition of IL-37b further elevated its expression (Fig. 4a). As reported, IL-1R8 will transfer onto the cell surface to bind with IL-37 upon stimulation^25^. Herein the surface expression and intracellular expression of IL-1R8 were measured. As shown in Fig. 4b, c, intracellular IL-1R8 remained unchanged in these groups. However, the expression of surface IL-1R8 was notably increased in KD-treated ECs, and IL-37b supplementation further upregulated surface IL-1R8 expression, which was consistent with the mRNA results, indicating that IL-37b might function via IL-1R8. To confirm this, HUVECs were transfected with empty plasmid (negative control) or IL-1R8 siRNA. Data showed that IL-1R8 siRNA remarkably silenced the expression of IL-1R8 (Fig. 4d). Moreover, the induced upregulation of IL-1R8 in KD-treated ECs was notably suppressed by IL-1R8 siRNA (Fig. 4e). The expression change of IL-1R8 was also substantiated at the protein level (Fig. 4f, g). Next, the effects of IL-1R8 silencing on EC apoptosis and inflammation were analyzed. As anticipated, the inhibition effect of IL-37b on apoptosis was significantly reversed upon IL-1R8 silencing, as demonstrated by increased percentage of TUNEL-positive cells and elevated BAX/Bcl-2 ratio (Fig. 4h–j). In addition, the decreased expression of TNF-α, IL-1β, and IL-6 mediated by IL-37b were also distinctly abolished by silencing of IL-1R8 (Fig. 4k). Taken together, these results provided strong evidence that IL-37b inhibited KD-treated EC apoptosis and inflammation via IL-1R8 pathway.

### Recombinant IL-37b inhibited ERK and NFκB activation in KD-treated ECs

**Fig. 5:**
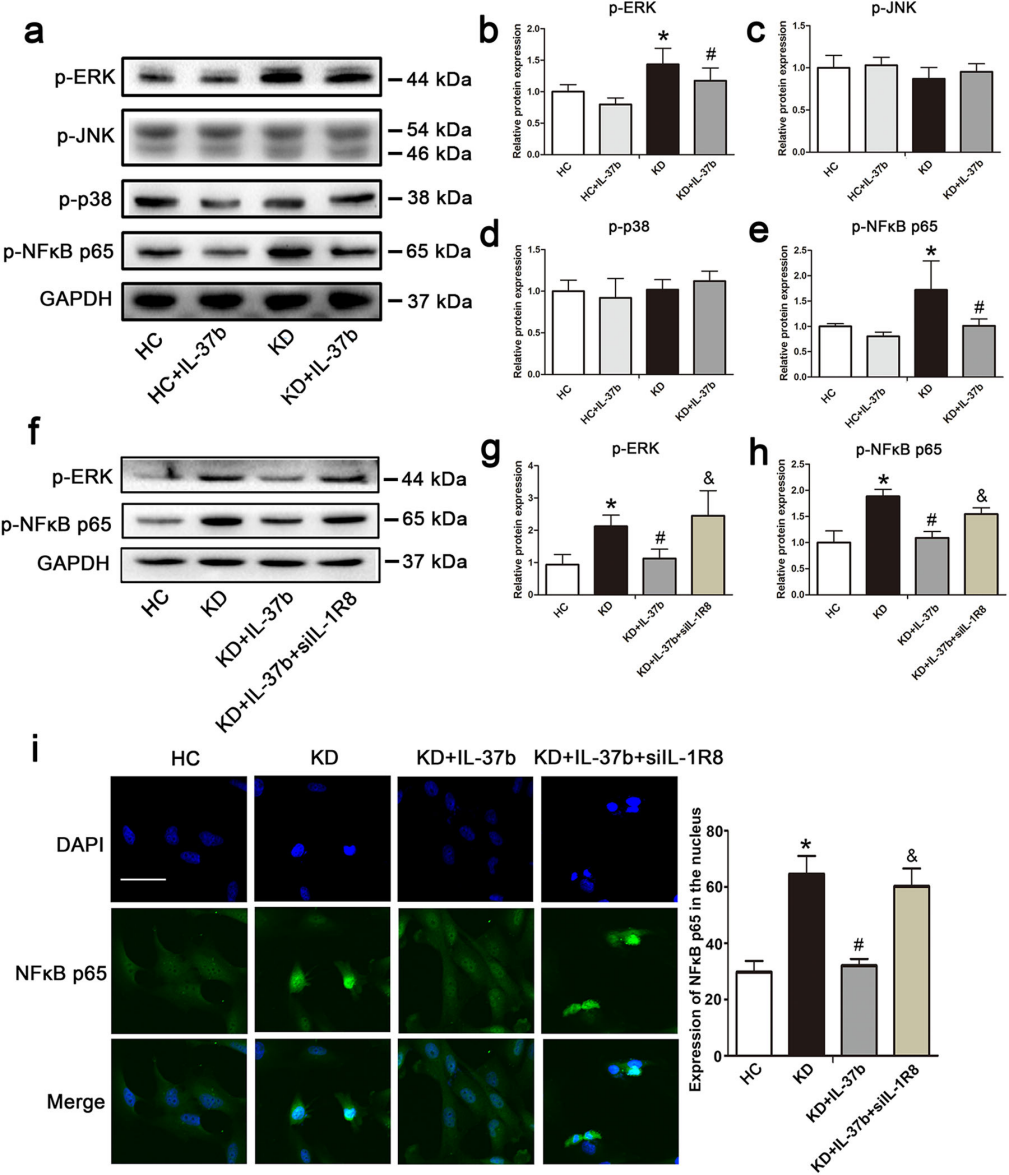
IL-37b inhibited the activation of ERK and NFκB in KD-treated ECs related to IL-1R8. **a** Effects of IL-37b treatment on the phosphorylation of ERK, JNK, p38, and NFκB p65 were assessed in the treated endothelial cells. **b**–**e** Quantitative analysis of these above proteins was conducted. Data are presented as mean ± SD (*n* = 3). Significance: *P < 0.05 vs. the HC group, and ^#^*P* < 0.05 vs. the KD group. **f**, **g** Effects of IL-1R8 silencing on the activation of ERK and NFκB p65 were observed. Significance: *P < 0.05 vs. the HC group, ^#^*P* < 0.05 vs. the KD group, and ^&^*P* < 0.05 vs. the KD + IL-37b group. **i** The nuclear translocation of NFκB p65 was observed after silencing IL-1R8 expression (*n* = 3). Magnification: ×200, Scale bar = 100 μm. Experiments were done at least three times in triplicate.

The intracellular signal transduction network showed that IL-37 can mediate a decrease in the components of the NFκB pathway, including p65^27^. Studies reported that IL-37b can inhibit mitogen-activated protein kinase (MAPK), including p38, c-Jun N-terminal kinase (JNK), and ERK, and NFκB p65 activation in response to IL-1β related to IL-1R8^9^. As is known, MAPK and NFκB signaling pathways are able to modulate cell apoptosis and inflammation^28^. Therefore, we first examined the activation of ERK, JNK, p38, and NFκB p65. As shown in Fig. 5a–e, ERK and NFκB p65 were significantly phosphorylated and activated after KD sera treatment. However, addition of IL-37b remarkably decreased the phosphorylation levels of ERK and NFκB p65, indicating that IL-37b could suppress the activation of these two proteins. To further substantiate that IL-37b modulated the activation of ERK and NFκB p65 via IL-1R8, IL-1R8 was silenced. As expected, a decrease in IL-1R8 expression significantly reversed the phosphorylation levels of ERK and NFκB p65 (Fig. 5f–h). Immunofluorescence staining showed that NFκB p65 translocation into the nucleus was attenuated upon IL-37b treatment. However, the translocation of NFκB p65 into the nucleus was enhanced after silencing of IL-1R8 compared with the IL-37b treatment group (Fig. 5i), further disclosing that IL-37b suppressed the activation of NFκB. Together, these data indicate that IL-37b treatment can affect the activation of ERK and NFκB.

### Recombinant IL-37b attenuated EC apoptosis and inflammation in a KD mouse model

**Fig. 6:**
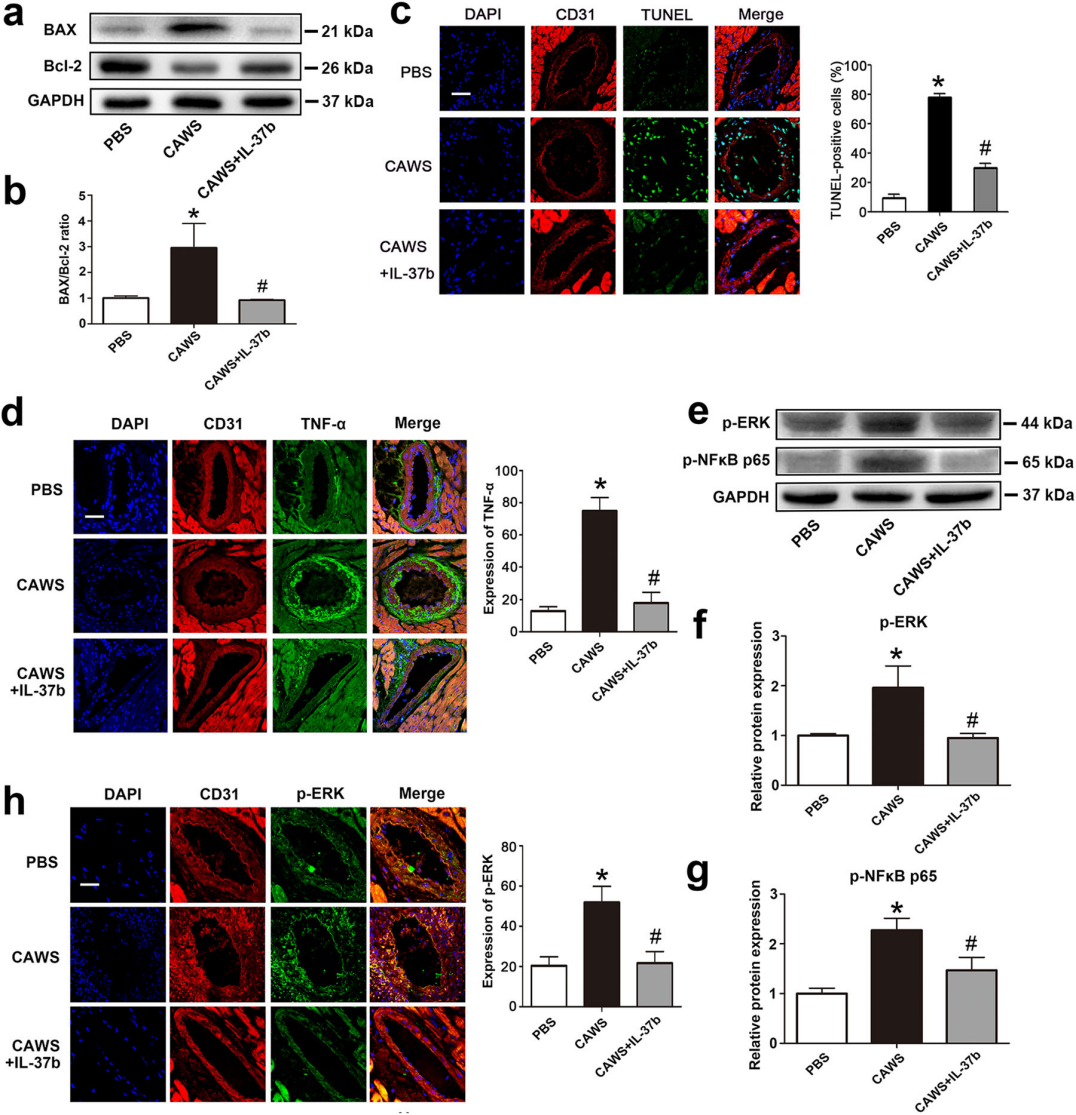
Endothelial cell apoptosis and inflammation were alleviated in a KD mouse model after IL-37b treatment. **a**, **b** The protein expression of BAX and Bcl-2 was examined in the KD mouse model after IL-37b treatment (*n* = 6). **c** Effects of IL-37b treatment on DNA fragmentation in the endothelial cells were evaluated by co-localized staining of TUNEL (green) and CD31 (an endothelial marker, red) (*n* = 5). Magnification: ×200. Scale bar = 50 μm. **d** TNF-α expression levels were examined in the coronary artery endothelial cells by double staining of TNF-α (green) and CD31 (red) (*n* = 5). Magnification: ×200. Scale bar = 50 μm. **e**–**g** The activation of ERK and NFκB was determined in the heart tissues by western blot analysis (*n* = 6). **h** The levels of p-ERK were examined in the coronary artery endothelial cells using p-ERK/CD31 double staining (*n* = 5). Magnification: ×200. Scale bar = 50 μm. Significance: *P < 0.05 vs. the PBS group, and ^#^*P* < 0.05 vs. the CAWS group. Each experiment was conducted at least three times.

To further explore the in vivo protective mechanism of IL-37b, the expression levels of apoptosis-associated proteins BAX and Bcl-2 were examined in the heart tissue from the KD mouse model. As shown in Fig. 6a, b, the ratio of BAX/Bcl-2 was significantly decreased upon recombinant IL-37b treatment. To further substantiate the effect on apoptosis occurring in coronary artery endothelium, CD31/TUNEL double staining was conducted. As shown in Fig. 6c, the percentage of TUNEL-positive cells in coronary artery endothelium of the KD mouse model was markedly reduced by IL-37b. In addition, the expression of TNF-α and IL-18 in the endothelium of coronary artery was also remarkably downregulated by IL-37b, as evidenced by decreased immunofluorescence intensity after CD31/TNF-α or CD31/IL-18 double staining (Fig. 6d and Fig. S2e). Moreover, the elevated plasma concentrations of TNF-α, IL-1β, IL-6, and IL-18 in the KD mouse model were also declined by treatment with IL-37b (Fig. S2a–d). These results indicated that IL-37b treatment significantly alleviated EC apoptosis and inflammation in the KD mouse model. To further demonstrate the mechanism of IL-37b inhibiting apoptosis and inflammation, the activation of ERK and NFκB p65 was analyzed. As shown in Fig. 6e–g, IL-37b treatment markedly decreased their phosphorylation level, and the immunofluorescence intensity of p-ERK was notably attenuated (Fig. 6h), indicating that IL-37b might mitigate endothelium damage via decreasing ERK and NFκB activation.

### Recombinant IL-37b mitigated coronary endothelium damage via IL-1R8 pathway in the KD mouse model

**Fig. 7:**
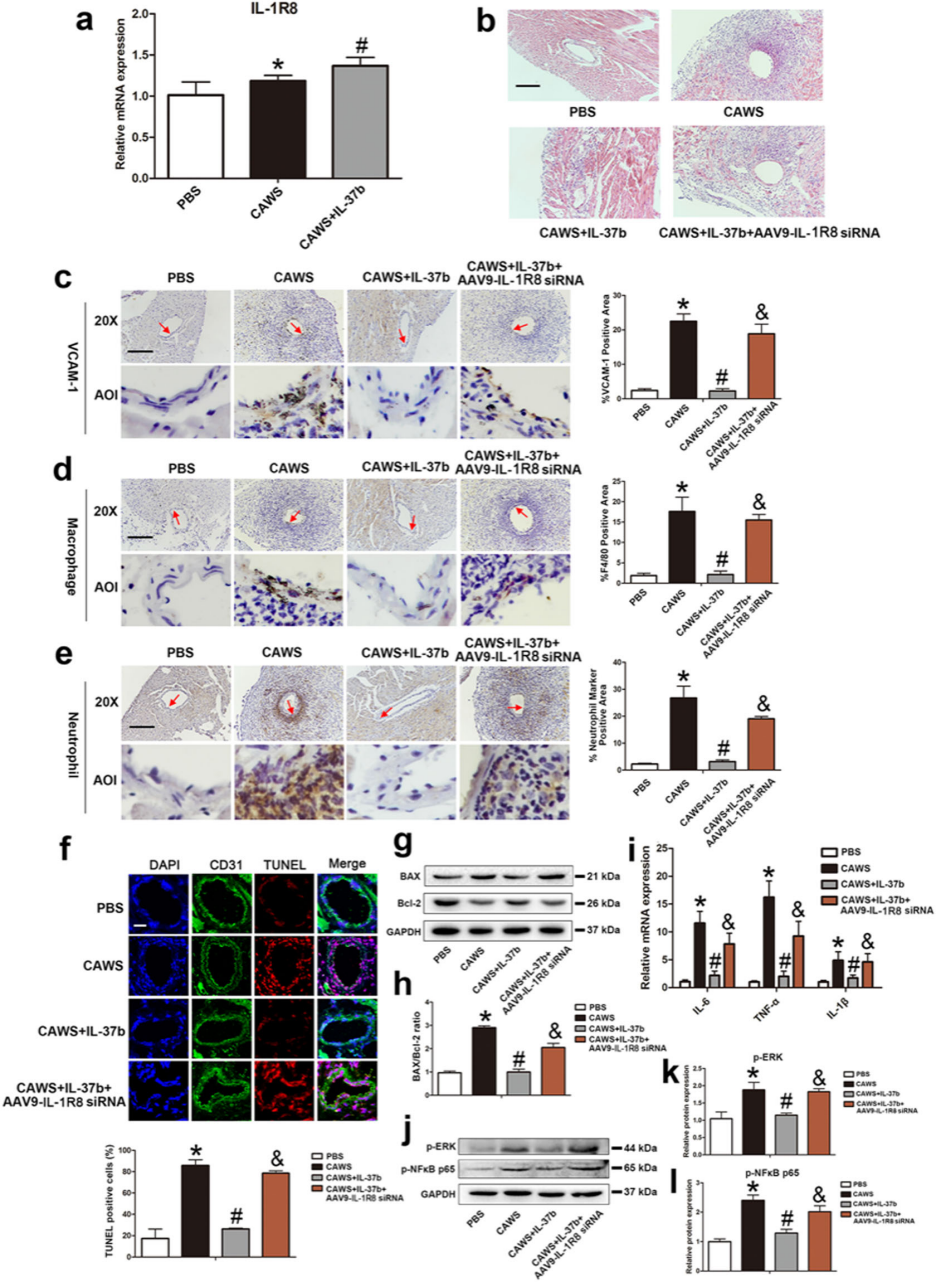
Mitigation of IL-37b-mediated coronary artery endothelial cell apoptosis and inflammation was realized via IL-1R8 pathway. **a** The expression of IL-1R8 were detected in the KD mouse model after IL-37b treatment (*n* = 6). **b** Effects of silencing of IL-1R8 on coronary arteritis were observed by H&E staining. Magnification: ×200. Scale bar = 100 μm. **c**–**e** The endothelial expression of VCAM-1, macrophage marker F4/80, and neutrophil marker was examined after IL-1R8 gene silencing using IHC staining. AOI were indicated with a red arrow. Scale bar = 100 μm. Right: The histograms respectively exhibited the percentage of VCAM-1, F4/80, and neutrophil marker-positive areas. Significance: *P < 0.05 vs. the PBS group, ^#^*P* < 0.05 vs. the CAWS group, and ^&^*P* < 0.05 vs. the CAWS + IL-37b group. **f** DNA fragmentation was examined using TUNEL staining. Scale bar = 100 μm. Below: Percentage of TUNEL-positive cells was shown in the histogram. Significance: *P < 0.05 vs. the PBS group, ^#^*P* < 0.05 vs. the CAWS group, and ^&^*P* < 0.05 vs. the CAWS + IL-37b group. **g**, **h** BAX/Bcl-2 ratio was analyzed by western blot analysis. **i** The mRNA expression levels of IL-6, TNF-α, and IL-1β were determined after silencing of IL-1R8. **j**–**l** Effects of IL-1R8 silencing on the phosphorylation level of ERK and NFκB p65 were assessed by western blotting. Significance: *P < 0.05 vs. the PBS group, ^#^*P* < 0.05 vs. the CAWS group, and ^&^*P* < 0.05 vs. the CAWS + IL-37b group. *N* = 6 mice per group, and each experiment was conducted at least three times.

To in vivo demonstrate whether IL-37b contributed to anti-apoptosis and anti-inflammation functions through IL-1R8 receptor in KD mouse model, the expression level of IL-1R8 was first determined. Consistent with in vitro results, the mRNA level of IL-1R8 was significantly upregulated in the CAWS group, and addition of IL-37b further increased its expression (Fig. 7a). To assess the role of IL-1R8 in vivo, an adeno-associated virus 9-mediated RNA interference targeting IL-1R8 (AAV9-IL-1R8 siRNA) was administrated into the KD mouse model in the presence of IL-37b. As shown in Fig. 7b, the inhibition effect of IL-37b on coronary inflammation was remarkably abrogated by IL-1R8 gene silencing. Furthermore, silencing of IL-1R8 also upregulated the expression of VCAM-1 and increased infiltration of macrophages and neutrophils into the endothelium compared with the IL-37b addition group (Fig. 7c–e). To further elucidate the effect of IL-1R8 on EC apoptosis and inflammation in vivo, apoptosis-related parameters and the expression of inflammatory cytokines were determined. Compared with the IL-37b treatment group, the percentage of TUNEL-positive cells in the endothelium and BAX/Bcl-2 ratio were notably increased upon silencing of IL-1R8 (Fig. 7f–h). Moreover, the downregulation of IL-6, TNF-α, and IL-1β mediated by IL-37b were also affected by IL-1R8 gene silencing (Fig. 7i). In addition, the phosphorylation levels of ERK and NFκB p65 were increased (Fig. 7j–l).

## Discussion

**Fig. 8:**
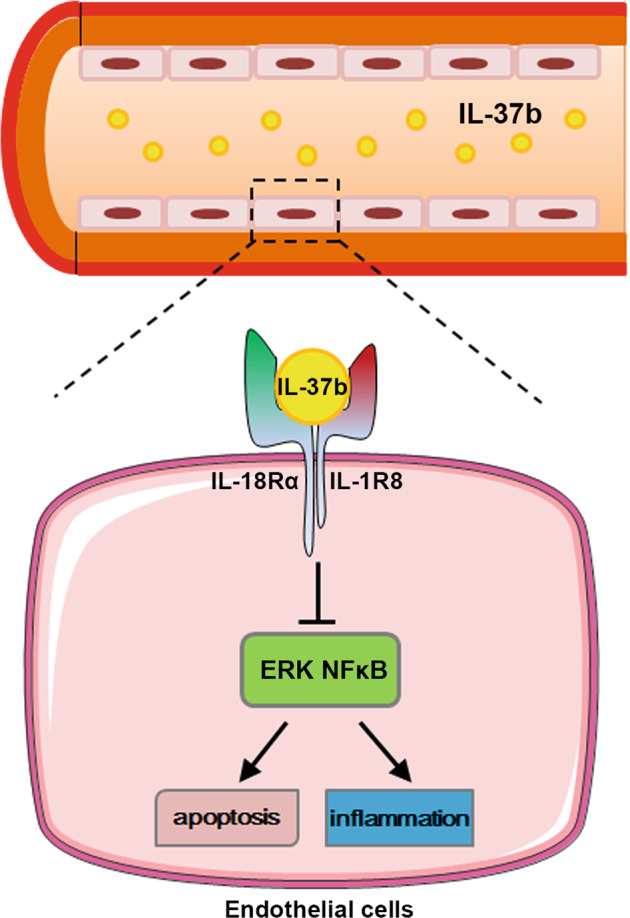
Schematic model for IL-37b against endothelial cell damage in Kawasaki disease. IL-37b binds to IL-18α and IL-1R8, inhibits ERK and NFκB activation, and finally suppresses EC apoptosis and inflammation.

In the current study, we revealed that IL-37b plays a critical protective role against KD and that the protective effect is realized via IL-1R8 pathway, inhibiting ERK and NFκB activation, which results in suppression of EC apoptosis and inflammation (Fig. 8). This previously unappreciated anti-inflammatory cytokine provides new clues to develop potential diagnosis, evaluation, and treatment strategies against KD.

KD is a kind of acute systemic vasculitis that involves severe inflammatory environment, which is caused by a variety of cells, including ECs. Therefore, targeting endothelial inflammation in KD could be a potential therapeutic strategy. IL-37 has emerged as an effective inhibitor to attenuate inflammation in models of osteoarthritis^9^, colitis^29^, and periodontal inflammation^30^. However, the level of IL-37 and whether it can have an effect on KD are still unidentified.

In this study, we first demonstrated that the level of IL-37 in sera from KD patients was significantly decreased compared with HCs. As is known, IL-37 has five spiced variants (IL-37a–e)^31^. Our qRT-PCR analysis showed that the expression of *IL37b* was significantly downregulated in KD serum-treated ECs, which was also substantiated by western blot analysis. However, the mRNA expression level of *IL37d* was elevated compared with HC serum-treated ECs. Studies reported that IL-37d is a functional cytokine that can negatively modulate the expression of pro-inflammatory cytokines and possesses anti-inflammatory roles^12,32^. Upregulation of IL-37d expression in KD serum-treated ECs might be a compensatory response since its elevation did not rescue EC inflammation mediated by KD serum. IL-37b was widely reported to have an anti-inflammatory effect^31^. Our in vivo study also demonstrated that the administration of recombinant human IL-37b significantly inhibited coronary artery inflammation and VCAM-1 expression in the KD mouse model, indicating that IL-37b inhibited EC activation and played a protective role in the pathophysiology of KD. As is known, the innate and adaptive immunity is activated during the acute phase of KD, resulting in an increase in cytokine production by immune effector cells. The secreted cytokine damage vascular ECs and induce the expression of leukocyte adhesion molecules on vascular endothelium, finally leading to infiltration of inflammation cells and coronary arteritis^33,34^. Therefore, the protective role of IL-37b can be explained by the following assumptions. One assumption is that IL-37b improves the inflammation microenvironment in KD, which then alleviates cytokine-mediated vascular endothelial injury and subsequent inflammatory cell recruitment. Another assumption is that IL-37b directly rescues damaged vascular ECs and decreases its inflammatory response and then mitigates the immune cell recruitment and coronary inflammation in KD. Both assumptions point out that rescuing damaged ECs is essential for IL-37b to inhibit coronary arteritis in KD.

Our following examination showed that IL-37b remarkably inhibited KD sera-induced EC apoptosis and inflammation. The anti-inflammatory activity of IL-37b has been reported in endometriosis^35^ and temporomandibular joint inflammation^9^. However, our study gave evidence for the first time that IL-37b has an anti-apoptosis effect in addition to anti-inflammatory activity. Zhang et al. reported that IL-37 reduces high glucose-induced inflammation and apoptosis of podocytes via inhibiting the signal transducer and activator of transcription factor 3–Cyclophilin A (CypA) signaling pathway^24^. Studies have demonstrated that the recombinant IL-37 precursor binds to immobilized IL-1R8 and IL-1R8 acts as the coreceptor for IL-37, and IL-37 fails to suppress LPS-induced cytokines as well as MAPK in dendritic cells from IL-1R8-deficient mice^25^. To clarify the mechanism of IL-37b-suppressing apoptosis and inflammation of KD-treated ECs, the expression of IL-1R8 receptor was examined. Data showed that the expression of IL-1R8 was significantly upregulated in KD-treated ECs, and IL-37b supplementation further elevated its expression. Moreover, the level of surface IL-1R8 was also accordingly changed. Further study exhibited that silencing of IL-1R8 abolished the inhibition effect of IL-37b on apoptosis and inflammation, indicating that IL-37b functioned via IL-1R8 pathway.

As is known, recombinant IL-37 binds to the immobilized ligand binding α-chain of the IL-18 receptor (IL-18Rα) as well as to the receptor IL-1R8 to mediate anti-inflammatory activity^25^. Studies reported that IL-18Rα is also required for the anti-inflammatory activity of IL-37 in addition to IL-1R8^27^. Therefore, we also examined the effect of IL-18Rα on IL-37b-mediated EC apoptosis and inflammation. Data showed that the addition of recombinant human IL-37b significantly inhibited KD serum-induced IL-18Rα upregulation. Moreover, overexpression of IL-18Rα reversed the suppression of IL-37b-regulated EC apoptosis and inflammation (Fig. S1). Our results were inconsistent with previous reports, in which mRNA levels of IL-18Rα were similar for LPS as well as LPS plus IL-37 in M1 macrophages^25^, indicating that IL-37 addition has no effect on LPS-induced IL-18Rα expression. This discrepancy might result from different cell types and distinct stimuli. In addition, studies reported that silencing of IL-18Rα results in a seemingly paradoxical increase in inflammation, which is attributed to the weakened association with IL-1R8^36^. However, in our present study, overexpression of IL-18Rα aggravated apoptosis and inflammation, which could be explained by the fact that IL-18 binds IL-18Rα, inducing the recruitment of IL-18Rβ to form a high affinity receptor, finally triggering a pro-inflammatory signal into the cells terminating in NFκB activation^37^. These results indicate that IL-18Rα can result in EC apoptosis and inflammation in KD.

In KD, the signaling cascades involving NFκB, and JNK and ERK MAPKs were activated^38^. Li et al. reported that IL-37 inhibits LPS-induced inflammation in macrophage via reducing the activation of p38, ERK, and JNK^25^. Another study demonstrated that IL-37 suppresses inflammation via inhibiting NFκB and ERK1/2 signaling pathway^39^. Luo et al. revealed that IL-37b can inhibit p38, ERK, JNK, and NFκB p65 activation in response to IL-1β^9^. The three MAPK protein kinases and NFκB are reported to participate in regulating apoptotic process and inflammatory response^40–42^. Therefore, we examined the phosphorylation level of ERK, JNK, p38, and NFκB p65. Results displayed that ERK and NFκB p65 were activated after KD sera treatment, which were suppressed after IL-37b addition. NFκB p65 translocation observation further demonstrated that IL-37b could inhibit NFκB p65 activation, suggesting that IL-37b might suppress EC apoptosis and inflammation via downregulating ERK and NFκB signaling pathway.

According to the above analysis, our data provide brand new evidence that IL-37b has a markedly protective effect on KD. Our results demonstrate that IL-37b addition can inhibit EC apoptosis and inflammation via the receptor IL-1R8, which causes inactivation of ERK and NFκB, finally alleviating KD coronary arteritis. Given that KD is clinically treated mainly through IVIG plus aspirin, which is expensive and still at risk to develop CAA exploring new alternative treatment drugs or strategies would be extremely valuable to the management of KD patients. Our new findings suggest that IL-37b may be a good candidate for inhibiting EC damage in KD. This exciting new opportunity awaits further deep research and subsequent clinical translation. However, some limitations exist in our present study. Whether other IL-37 variants also function in KD remains uncertain and requires further investigation. Nevertheless, our current study exhibits novel evidence that IL-37b is a very effective candidate drug to treat KD.

## Materials and methods

### Patients’ blood sample and ethical consideration

Collection of patients’ blood samples were performed according to our previous report^7^. All the harvested sera samples were regularly stored at −80 °C within 4 h after collection for later use. Moreover, all the participants gave written informed consent for using their clinical information and blood samples for academic research. This research was authorized by the ethics committee of Wenzhou Medical University, and conducted in accordance with the Helsinki Declaration.

### Enzyme-linked immunosorbent assay

Serum concentrations of IL-37 were determined by the corresponding ELISA kits according to the manufacturer’s instructions. ELISA kit for human IL-37 was purchased from Solarbio (Beijing, China).

### Quantitative real-time polymerase chain reaction

Cells and/or tissues were harvested and lysed in Trizol. Total RNA was collected and reverse-transcribed using a PrimeScript™ RT Reagent Kit with gDNA Eraser (Takara) to obtain cDNA. Next, real-time PCR was performed on Applied Biosystems QuantStudio 3 real-time PCR system (ThermoFisher) with 40 cycles using Power TB green PCR Master Mix (Takara). Glyceraldehyde 3-phosphate dehydrogenase (GAPDH)-specific primers were used as an internal control. The expression of all the target genes was normalized to GAPDH, and the relative change of gene expression was calculated using 2^−ΔΔCT^ method. The primers used in this study are shown in Table 1.

**Table 1:** QRT-PCR primers used in this study.

Gene	Forward primer (5’→3’)	Reverse primer (5’→3’)
GAPDH	AAGAAGGTGGTGAAGCAGG	GAAGGTGGAAGAGTGGGAGT
IL-1β	GAAATGATGGCTTATTACAGTGGCA	GTAGTGGTGGTCGGAGATTCGTAG
TNF-α	CTTGTTGCCTCCTCTTTTGCTTA	CTTTATTTCTCTCAATGACCCGTAG

### Western blot analysis

The heart and HUVECs were collected and lysed using protein extraction reagents. Then the complex was centrifuged at 12,000 rpm for 20 min at 4 °C, and the supernatant was quantified with BCA reagents. The obtained proteins were separated on a 10 or 12% gel and transferred onto a polyvinylidene difluoride membrane. After blocking with 5% skimmed milk in TBST (TBS with 0.05% tween 20) for 2 h, membranes were incubated with the following primary antibodies at 4 °C overnight: IL-37 (Proteintech, Chicago, USA, 1:1000, Cat. No: 60296-1-lg), IL-1R8 (Proteintech, Chicago, USA, 1:1000, Cat. No: 27828-1-AP), ATP1A1 (Proteintech, Chicago, USA, 1:10,000, Cat. No: 14418-1-AP), p-ERK (Affinity Biosciences, Cincinnati, OH, USA, 1:1000, Cat. No: AF1015), p-JNK (Affinity Biosciences, Cincinnati, OH, USA, 1:1000, Cat. No: AF3318), p-p38 (Affinity Biosciences, Cincinnati, OH, USA, 1:1000, Cat. No: AF4001), p-NFkB p65 (Affinity Biosciences, Cincinnati, OH, USA, 1:1000, Cat. No: AF2006), BAX (Signalway Antibody, California, USA, 1:1000, Cat. No: 40635), Bcl-2 (Signalway Antibody, California, USA, 1:1000, Cat. No: 40639), and GAPDH (Proteintech, Chicago, USA, 1:2000, Cat. No: 60004-1-lg). After washing with TBST for three times, the membranes were treated with horseradish peroxidase (HRP)-conjugated secondary antibodies (1:10,000) for 2 h at room temperature. Signals were visualized by ChemiDoc XRS + Imaging System (Bio-Rad Laboratories, Hercules, CA, USA). The densitometric values of bands on western blot were obtained by the Image J software and were subjected to statistical analysis.

### Preparation of CAWS

As reported before, CAWS were prepared using *C. albicans* strain NBRC1385^16,43^. In brief, *C. albicans* were cultured in C-limiting medium at 27 °C for 2 days at a rotation speed of 170 rpm. Next, equal volume of ethanol was added and kept in the refrigerator at 4 °C overnight. After that, the cultures were collected by centrifugation, and the pellets were dissolved in water and stirred for 2 h followed by centrifugation again. Next, the soluble part was obtained, mixed with equal volume of ethanol at 4 °C overnight, and centrifuged again to obtain the pellets. Finally, the pellets were collected by acetone and kept undisturbed overnight. The obtained CAWS were dissolved in phosphate-buffered saline (PBS) buffer and autoclaved before use.

### Animals and experimental design

For animal models of KD, male C57BL/6 mice at the age of 3–4 weeks were purchased from Wenzhou Medical University, License No. SCXK[ZJ]2005-0019. All experimental procedures for animal studies were approved by the ethics committee of Wenzhou Medical University and performed in accordance with the Guide for the Care and Use of Laboratory Animals. Animals were housed at 23 ± 2 °C with humidity of 50 ± 5%. Mice were randomly divided into four groups (*n* = 6 for each group): PBS group, CAWS group, CAWS + IL-37b group, CAWS + IL-37b + AAV9-IL-1R8 siRNA group. The mice in the CAWS (4 mg/body) treated-group were injected intraperitoneally once a day for 5 days, while mice in the PBS group were injected with PBS buffer. Recombinant protein IL-37b (40 μg/kg body weight) was intraperitoneally administered into mice 2 days before and after CAWS addition. After 4 weeks of final CAWS injection, the mice were anesthetized and sacrificed for collection of heart tissues and follow-up examinations.

### Hematoxylin and eosin (H&E) staining and IHC staining

Mice hearts were obtained and then fixed with 4% paraformaldehyde and embedded with paraffin. Five-µm-thick heart sections were dewaxed and hydrated, then stained with H&E solution, and observed under light microscope. IHC staining of F4/80, neutrophil, and VCAM-1 was performed as follows. Sections were dewaxed and hydrated, then incubated in 3% H_2_O_2_ for 15 min. After blocking with 5% bovine serum albumin (BSA) at 37 °C for 30 min, primary antibody was incubated overnight at 4 °C. Next, HRP-conjugated secondary antibodies were used for detection. Diaminobenzidine was used as substrate for color development. All slides were counterstained with hematoxylin. Images of histologically stained sections were obtained using light microscope.

### Cells culture and treatment

In this study, HUVECs and human monocytic leukemia cell line, THP1, were utilized. These cell lines were purchased from American Type Culture Collection (ATCC, Manassas, VA, USA) and authenticated by the Genetic Testing Biotechnology Corporation (Suzhou, China) and the KeyCen BioTech (Nanjing, China) through short tandem repeat markers. There was no mycoplasma contamination. HUVECs and THP1 were routinely cultured as reported before^16^. In the coculture system, HUVECs were cultured in the lower chamber, and THP1 cells were placed in the upper chamber that permits diffusion of soluble molecules. Then KD or HC sera were added to the upper chamber and treated THP1 cells for 24 h. At the same time, the HUVECs in the lower chamber would be affected by the diffusing molecules. All cultures were grown in Dulbecco’s modified Eagle’s medium (DMEM). If necessary, the HUVECs would be pretreated with IL-37b protein (R&D Systems, Minneapolis, MN, USA) for 1 h and then co-cultured with THP1 cells for 24 h. All cultures were grown in HC serum or KD serum that was diluted in DMEM.

### Immunofluorescence staining

After dewaxing and hydration, the heart sections were incubated in 3% H_2_O_2_ for 15 min and then blocked with 5% BSA at 37 °C for 30 min. Subsequently, the sections were incubated with primary antibodies against p-ERK, TNF-α, and CD31 overnight at 4 °C followed by incubation at 37 °C with Alexa Fluor 647 or Alexa Fluor 488(1:1000, Abcam) for 1 h. For HUVECs, the cells were fixed in 4% paraformaldehyde (PFA) for 30 min and blocked with 5% BSA for 30 min at 37 °C. Then the cells were incubated with the primary antibody against NFκB p65 overnight at 4 °C. The Alexa Fluor 488 secondary antibody was applied at a 1:1000 dilution for 1 h. Nuclei were labeled with 4,6-diamidino-2-phenylindole (DAPI). The images were captured under confocal laser microscopy (Nikon, Tokyo, Japan).

### DNA fragmentation evaluation

TUNEL staining was used to detect DNA fragmentation of ECs. ECs were cultured on coverslips and treated with serum. After fixation with 4% PFA and permeabilization with 0.3% Triton X-100, cells were washed with PBS, incubated with TUNEL reaction mixture at 37 °C in the dark for 1 h, and stained by DAPI. Finally, the images were scanned and captured under confocal laser microscopy (Nikon, Tokyo, Japan).

### Statistical analysis

Every experiment was repeated at least three times, and data are expressed as mean ± SD. All the data were analyzed using SPSS version 17.0, and they were normally distributed. For statistical significance analysis, a two-tailed unpaired Student’s *t* test was performed to compare two experimental groups, and one-way analysis of variance test followed by Duncan’s multiple range test was conducted for the comparison of more than two groups. *P* values <0.05 were regarded as statistically significant. Sample size was chosen based on previous studies^14,15^, which conducted similar experiments to observe significant results. Variance was similar between the groups that were being statistically compared.

## Supplementary information

Supplementary information
