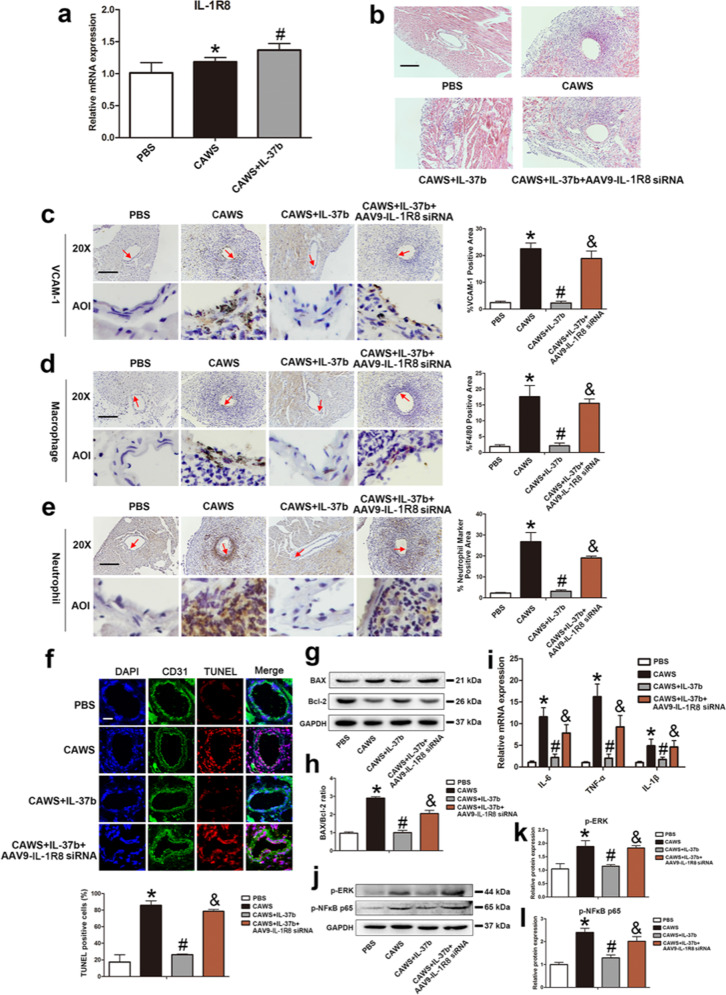# Correction: IL-37b alleviates endothelial cell apoptosis and inflammation in Kawasaki disease through IL-1R8 pathway

**DOI:** 10.1038/s41419-021-04044-5

**Published:** 2021-08-02

**Authors:** Chang Jia, Yingzhi Zhuge, Shuchi Zhang, Chao Ni, Linlin Wang, Rongzhou Wu, Chao Niu, Zhengwang Wen, Xing Rong, Huixian Qiu, Maoping Chu

**Affiliations:** 1 Pediatric Research Institute, The Second Affiliated Hospital and Yuying Children’s Hospital of Wenzhou Medical University, 325027 Wenzhou, China; 2 Children’s Heart Center, Institute of Cardiovascular Development and Translational Medicine, The Second Affiliated Hospital and Yuying Children’s Hospital of Wenzhou Medical University, 325027 Wenzhou, China

**Keywords:** Vasculitis, Drug development

Correction to: *Cell Death and Disease* 10.1038/s41419-021-03852-z, published online 3 June 2021

Since the publication of this paper, the authors have noted that there was an error in Fig. 7b–l. Mice were randomly divided into four groups: PBS group, CAWS group, CAWS +IL-37b group, CAWS +IL-37b +AAV9-IL-1R8 siRNA group. However, CAWS+IL-37b+AAV9-IL-37 siRNA was marked by mistake. This error has now been rectified. The correct figure is shown below. The original article has been corrected.