# Supplementary material for: IL-37b alleviates endothelial cell apoptosis and inflammation in Kawasaki disease through IL-1R8 pathway
Source: Cell Death Dis. 2021 Jun 3;12(6):575. doi: 10.1038/s41419-021-03852-z (PMC8174541; doi:10.1038/s41419-021-03852-z)
Supplement: Supplementary file 1 — Supplementary information [file 41419_2021_3852_MOESM1_ESM.docx]

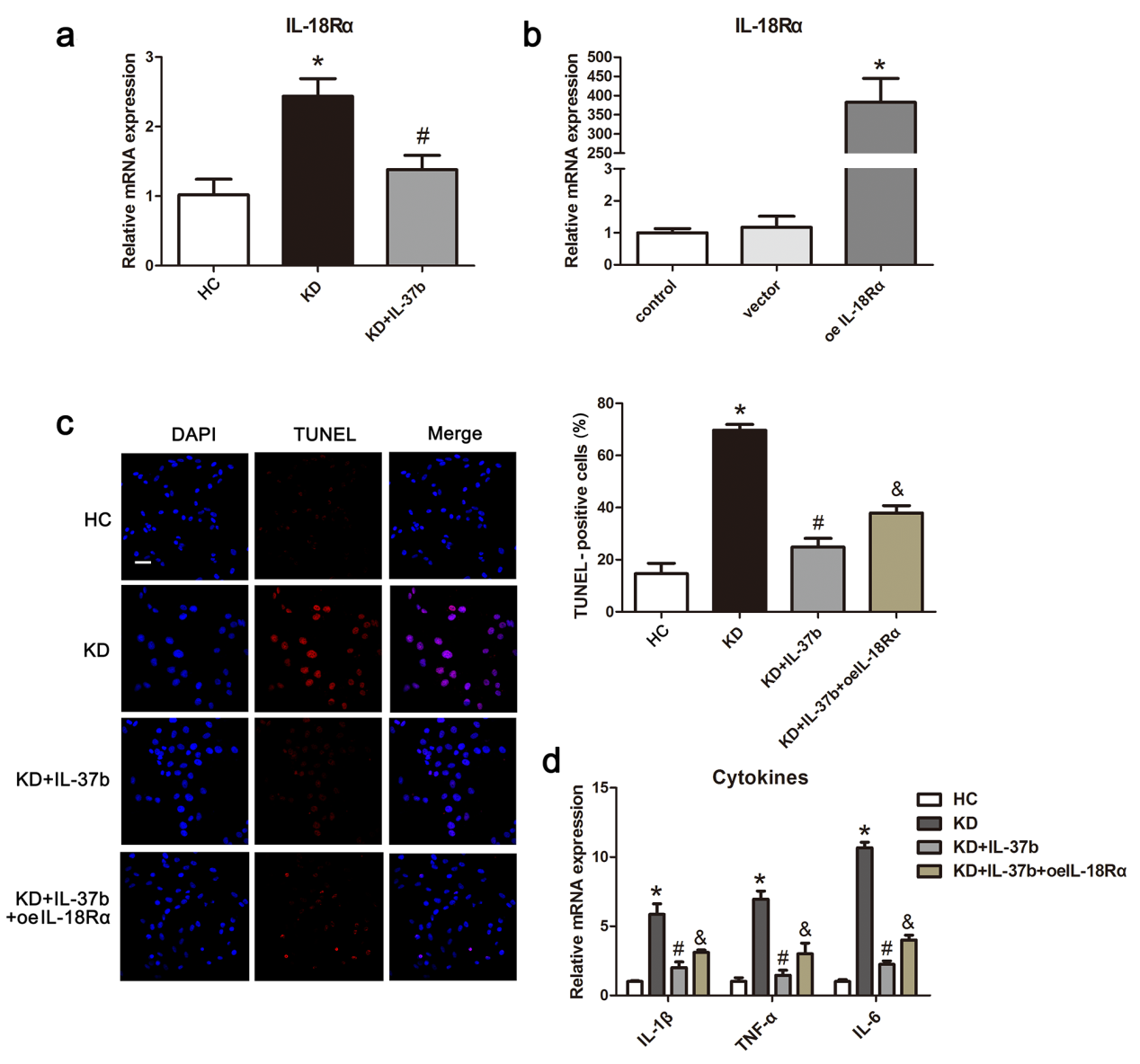


**Fig. S1 IL-37b mitigated endothelial cell apoptosis and inflammation associated with IL-18Rα. a** The mRNA expression of IL-18Rα was examined by qRT-PCR analysis (n=3). Significance: * *P*<0.05 vs. the HC group, and # P<0.05 vs. the KD group. **b** Endothelial cells were transiently transfected with vector plasmid (vector) or IL-18Rα overexpression plasmid (oeIL-18Rα). After that, the expression change of IL-18Rα was examined (n=3). **c** DNA fragmentation was analyzed using TUNEL staining after IL-18Rα was overexpressed (n=3). Significance：* *P*<0.05 vs. the HC group, # *P*<0.05 vs. the KD group, and & *P*<0.05 vs. the KD + IL-37b group. Magniﬁcation: × 200, Scale bar =100 μm. **d** The expression levels of cytokines were assessed after overexpressing IL-18Rα (n=3). Significance：* *P*<0.05 vs. the HC group, # *P*<0.05 vs. the KD group, and & *P*<0.05 vs. the KD + IL-37b group. All these experiments were done at least three times.


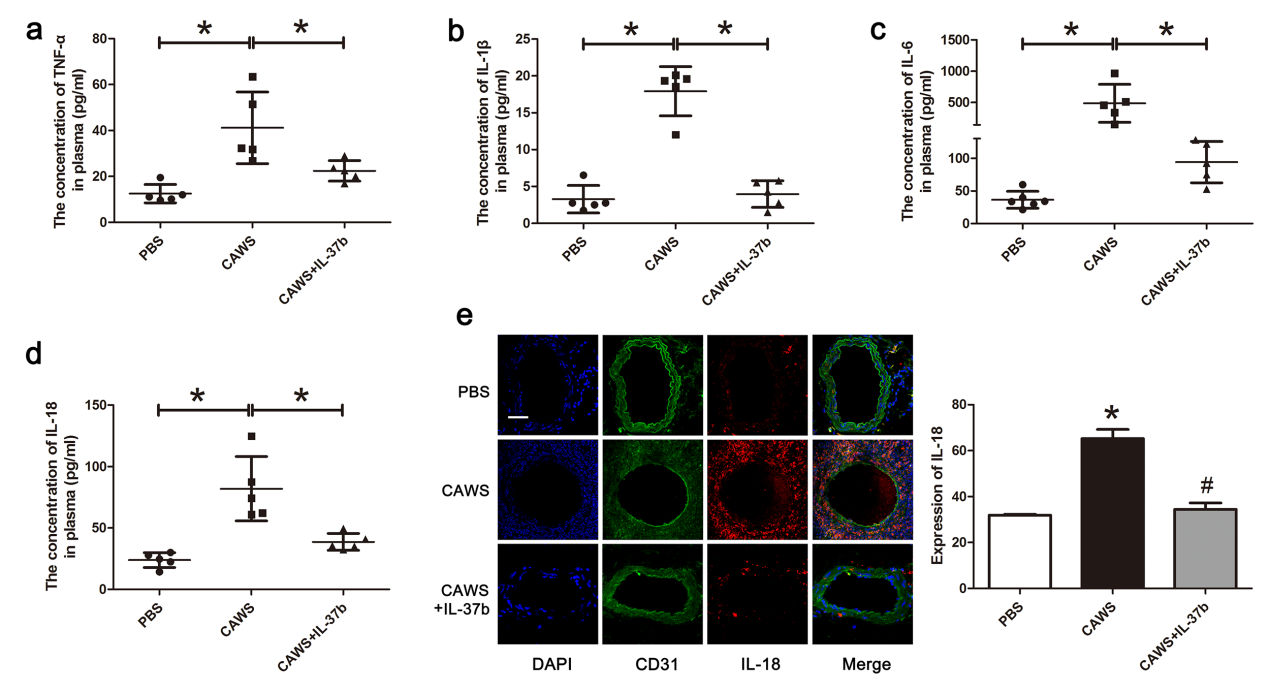


**Fig. S2 Levels of several cytokines were examined in the KD mouse model with or without treatment with IL-37b. a-d** The concentrations of TNF-α, IL-1β IL-6, and IL-18 were detected in the plasma of KD mice with or without IL-37b treatment by enzyme-linked immunosorbent assay (ELISA) (n=5). Significance: **P* < 0.05. **e** The expression of IL-18 was determined in coronary artery endothelial cells using double staining of IL-18 (red) and CD31 (green) (n=5). Magniﬁcation: ×200. Scale bar =50 μm. Significance: * *P* < 0.05 vs. the PBS group, and # *P* < 0.05 vs. the CAWS group. All these experiments were repeated at least three times.
